# Level of attendance at the English National Health Service Diabetes Prevention Programme and risk of progression to type 2 diabetes

**DOI:** 10.1186/s12966-023-01554-7

**Published:** 2024-01-12

**Authors:** Beth Parkinson, Emma McManus, Rachel Meacock, Matt Sutton

**Affiliations:** Health Organisation, Policy, and Economics (HOPE) Research Group, The Centre for Primary Care and Health Services Research, The University of Manchester, Manchester, UK

**Keywords:** Prevention, Type 2 Diabetes, Education and Behavioural Interventions, Prediabetes, Dose Response

## Abstract

**Background:**

We evaluated the dose–response relationship between the level of attendance at the English National Health Service Diabetes Prevention Programme (DPP) and risk of progression to type 2 diabetes amongst individuals participating in the programme.

**Methods:**

We linked data on DPP attendance for 51,803 individuals that were referred to the programme between 1st June 2016 and 31st March 2018 and attended at least one programme session, with primary care records of type 2 diabetes diagnoses from the National Diabetes Audit up to 31st March 2020. Weibull survival regressions were used to estimate the association between the number of programme sessions attended and risk of progression to type 2 diabetes.

**Results:**

Risk of developing type 2 diabetes declined significantly for individuals attending seven of the 13 programme sessions and continued to decline further up to 12 sessions. Attending the full 13 sessions was associated with a 45.5% lower risk (HR: 0.545 95% CI: 0.455 to 0.652). Compared to individuals that only partially attended the programme, attendance at 60% or more of the sessions was associated with a 30.7% lower risk of type 2 diabetes (HR: 0.693 95% CI: 0.645 to 0.745).

**Conclusions:**

Reducing the risk of progression to type 2 diabetes through diabetes prevention programmes requires a minimum attendance level at seven of the 13 programme sessions (54%). Retaining participants beyond this minimum level yields further benefits in diabetes risk reduction. Commissioners may wish to consider altering provider payment schedules to incentivise higher retention levels beyond 60% of programme sessions.

**Supplementary Information:**

The online version contains supplementary material available at 10.1186/s12966-023-01554-7.

## Background

The rising burden of long-term conditions has increased the focus on disease prevention and health promotion globally. Diabetes is of particular concern, with an estimated 8.6% of people (3.8 million) aged 16 and over in England having diabetes, and the prevalence is expected to increase to 9.7% (4.9 million) by 2035 [1]. Diabetes places an enormous burden on both patients and the health system, with an estimated cost to the National Health Service (NHS) of £9.8 billion a year, or 10% of the NHS budget [2]. The incidence of type 2 diabetes is largely preventable and randomised controlled trials in those at risk of developing type 2 diabetes have shown that onset can be prevented through behaviour change interventions [3, 4].

The NHS Diabetes Prevention Programme (DPP) was therefore developed in an attempt to prevent or delay the onset of type 2 diabetes amongst adults identified to be at high risk, defined as having non-diabetic hyperglycaemia [5]. The programme consists of at least 13 sessions lasting 1–2 h each spread across a minimum of 9 months. This is based on evidence from clinical trials of similar behavioural interventions, which suggested that programmes with these features were the most effective [6].

Emerging evidence suggests the NHS DPP is having a significant impact on outcomes and may be cost-effective even in the short-term [7]. Studies indicate that completion of the programme is associated with improvements in intermediate outcomes such as reductions in weight and HbA1c [8, 9]. The magnitudes of weight loss and HbA1c reductions were found to increase with the number of programme sessions attended [8]. A study using general practice records of people diagnosed with non-diabetic hyperglycaemia found that people who were recorded as being referred to the programme were 20% less likely to develop type 2 diabetes compared to those who were not referred to the programme [10].

However, these studies have not examined the extent to which the level of attendance at the programme is associated with the onset of type 2 diabetes. The time commitment involved in such programmes is a barrier to uptake and continued attendance for many individuals [11–13]. Indeed, uptake figures from the first 100,000 referrals to the NHS DPP show that there is substantial room for improvement in terms of retention in the programme, with around half of those referred not even making it to the initial assessment [14]. Among those that attended this initial assessment, 34% achieved the desired level of attendance (≥ 60% of sessions [8]), and 22% attended the full course [14]. Therefore, examining the level of attendance required to achieve a significant reduction in type 2 diabetes risk, and whether there is a level of attendance after which no further benefits accrue, could assist in designing a more achievable programme for people to complete.

We aimed to provide the first analysis of the association between DPP attendance and the risk of type 2 diabetes and investigated whether there is evidence of a dose–response relationship between an individual’s level of attendance and their risk of progression to type 2 diabetes.

## Methods

### Intervention

Individuals are eligible for the NHS DPP if they have been identified as having non-diabetic hyperglycaemia. This is defined by a blood test showing a concentration of glycated haemoglobin (HbA1c) of 42–47 mmol/mol (6.0–6.4%) or a fasting plasma glucose of 5.5–6.9 mmol/l. Once individuals are identified as eligible, they can be offered a place on the programme, which is usually done by their general practice, either following a consultation or by letter [14]. The practice must then gain consent from the individual for their referral to be passed on to the DPP providers. Receipt of this referral by the programme providers then generates a record in the DPP Minimum Data Set, which is the dataset we used to define our study cohort.

Following referral, participants are invited by the DPP providers to attend an initial assessment at which eligibility for the programme is confirmed, baseline weight measurements are taken, and additional participant characteristics recorded. Individuals are then invited to attend a series of face-to-face group intervention sessions. The programme consists of a minimum of 13 sessions, each lasting 1–2 h, spread across a minimum of 9 months. The intervention utilises behaviour change techniques such as goal setting, feedback, and self-monitoring with the aim of encouraging weight loss, improved nutrition and increased physical activity to enable individuals to reduce their risk of developing type 2 diabetes [15]. Whilst it was mandatory for all providers to cover certain topics as part of their programmes (including providing type 2 diabetes information, risk factors for type 2 diabetes, weight loss, and dietary and physical activity information), there was flexibility in the service specification with regards to the exact structure and content of the programme [15, 16].

There were four providers of the programme during our study period, three of which offered programmes totalling 13 intervention sessions, and one of which offered a programme of 18 intervention sessions in length. Whilst participants are encouraged to attend all sessions on their programme, missing a session does not prevent a participant from attending further sessions. The only exception is the initial assessment, which individuals must attend before accessing the group intervention sessions.

### Data sources

We utilised person-level data from two main sources: the DPP Minimum Data Set collected by programme providers, and the National Diabetes Audit extracted from general practice records. These datasets were linked at the individual level, using a pseudonymised linkage file provided by NHS Digital.

The DPP Minimum Data Set contains information on all referrals received by DPP providers. We examined referrals from the beginning of the programme on 1st June 2016 to 31st March 2018. DPP providers are contractually obliged to collect this data to receive financial reimbursement from NHS England.

The National Diabetes Audit data contains information extracted from general practice records on all individuals with a Read code [17] indicating a diagnosis of type 2 diabetes by the final date of the audit extract period, 31st March 2020.

T﻿his study is part of the DIPLOMA programme of research, which was reviewed and approved by the North West Greater Manchester East NHS Research Ethics Committee (Reference: 17/NW/0426, 1st August 2017). The analysis was undertaken using anonymised routinely collected healthcare data and informed consent from individuals was not necessary.

### Analysis sample

We restricted the analysis cohort to those that were referred up to 31st March 2018 (n = 182,335) to allow enough time for people to finish the programme, and for impacts on diabetes progression to be detected within the follow-up period. To examine the dose–response relationship, we focused on the individuals that attended at least one programme session following their initial assessment (*n* = 67,739). This focus enabled comparability with an existing study of the dose–response relationship between NHS DPP session attendance and the intermediate outcomes of weight and HbA1c change [8].

The median time between initial assessment and 60% programme completion is 345 days [9]. Continuing participation in the DPP requires that a person remains without a diagnosis of type 2 diabetes during this period. Therefore, participants that developed diabetes part-way through would not have had the opportunity to finish the full programme. In our main analysis, we therefore defined time at risk as starting 12 months after referral to avoid survivorship bias. As a result, we omitted 1,729 (2.5%) participants that developed diabetes within the 12 months following referral. We examined the sensitivity of our results to this exclusion by repeating the analysis with time at risk instead starting from the date of the first session attended and including individuals that converted to type 2 diabetes within 12 months from referral in this sensitivity analysis.

The exposure of interest in our main analysis was an indicator of the count of individual programme sessions a person attended. Rules around data disclosure mean that we cannot present any results which may allow the identification of individual providers. We therefore restricted this main analysis to participants at the three providers who offered programmes of 13 sessions in length (n = 51,803). We conducted supplementary analysis using a binary measure of exposure, which did not require the disclosure of the number of individual sessions attended, and therefore allowed us to examine the full sample of participants at all four providers (n = 66,010).

### Outcome

The outcome of interest was a diagnosis of type 2 diabetes in the National Diabetes Audit.

### Explanatory variables

The key exposure of interest was the level of programme attendance, which we examined in two different ways. The main analysis involved an indicator for the count of individual programme sessions a person attended. This variable can take 13 values indicating the total number of programme sessions each individual attended. We conducted this analysis on the main analysis sample of participants at the three providers who offered programmes of 13 sessions in length (*n* = 51,803).

Secondly, to allow us to assess individuals referred to all four providers (*n* = 66,010), we used a binary indicator for programme completion, defined by NHS England as attending at least 60% of sessions. Completion is equivalent to attending at least 8 sessions for the three providers whose programme consists of 13 sessions, and at least 11 sessions for the remaining provider whose programme consists of 18 sessions. This was compared to the reference category of partial attendance, defined as < 60% of programme sessions.

The Minimum Data Set contained several participant characteristics which we used in our analyses: age at referral, sex, area of residence, the general practice the participant was registered with, ethnicity, body mass index (BMI) at initial assessment, employment status, smoking status, and disability. Area of residence was classified in terms of lower-layer super output areas (LSOAs), of which there are 32,844 in England with a mean population of 1,500 in each [18]. We grouped age at referral date into seven age bands. We merged information on the deprivation level in the area in which the individual resided using the indices of multiple deprivation [19]. We also linked general practice code to the NHS England commissioning region to control for regional differences in programme provision [20]. To avoid loss of data, we used the missing indicator method to create a ‘missing’ category for each categorical variable.

### Statistical analyses

We used parametric survival models with a Weibull survival distribution to estimate the association between the level of attendance at the NHS DPP and developing type 2 diabetes. A Weibull survival distribution was used as this minimised the Akaike Information Criterion compared to models with either exponential or log-normal distributions. Separate regressions were run for the 13-category indicator for the number of programme sessions attended, and a binary indicator for programme completion (≥ 60% of sessions). For the analyses which used a binary indicator of programme completion, we also ran this first for the sample of participants at the three providers who offered programmes of 13 sessions in length (n = 51,803) as in the main analysis, and then the full sample of participants at all four providers (n = 66,010),

We controlled for age group, sex, ethnicity, BMI, employment status, smoking status, disability, the level of deprivation in the area in which the individual resided, the DPP provider and the NHS England region. We also included the month-year of referral to control for the length of time since the programme was initially implemented.

#### Supplementary analysis

We examined the sensitivity of the results from the main analysis to the exclusion of participants who may not have had the opportunity to finish the programme because they developed type 2 diabetes within 12 months of their referral. For this supplementary analysis, we defined time at risk from the date of the first session.

We also examined the sensitivity of the results to the assumption of proportional hazards in the Weibull model. Preliminary analysis using Cox proportional hazards regressions and evaluation of the proportional hazards assumption using Schoenfeld residuals showed some evidence of non-proportional hazards. Therefore, as a further supplementary analysis, we repeated the main analysis using accelerated failure time models [21].

## Results

### Descriptive statistics

Table 1 displays descriptive statistics for participant characteristics recorded at the start of the programme, for the full sample of participants in column one, and then separately for individuals that only partially attended the programme (< 60% of sessions) and those that completed the programme according to the NHS England definition of completion (≥ 60% of sessions).

**Table 1:** Descriptive statistics—Participant characteristics by level of programme attendance

	All participants		Partial attendance (< 60% sessions)		Completed programme (≥ 60% sessions)	
	*N* = 51,803		*N* = 25,015		*N* = 26,788	
Age category						
Aged 18 to 34	547	1.1%	419	1.7%	128	0.5%
Aged 35 to 44	2294	4.4%	1597	6.4%	697	2.6%
Aged 45 to 54	6410	12.4%	3906	15.6%	2504	9.3%
Aged 55 to 64	12,396	23.9%	6168	24.7%	6228	23.2%
Aged 65 to 74	19,231	37.1%	7847	31.4%	11,384	42.5%
Aged 75 to 84	9648	18.6%	4364	17.4%	5284	19.7%
Aged 85 +	1277	2.5%	714	2.9%	563	2.1%
Sex						
Female	28,258	54.5%	13,604	54.4%	14,654	54.7%
Male	23,545	45.5%	11,411	45.6%	12,134	45.3%
Deprivation quintile						
Most deprived	8301	16.0%	4751	19.0%	3550	13.3%
2	9291	17.9%	4702	18.8%	4589	17.1%
3	10,991	21.2%	5107	20.4%	5884	22.0%
4	10,988	21.2%	5001	20.0%	5987	22.3%
Least deprived	12,176	23.5%	5426	21.7%	6750	25.2%
Missing	56	0.1%	28	0.1%	28	0.1%
BMI						
Under/healthy weight	8107	15.6%	3596	14.4%	4511	16.8%
Overweight	18,612	35.9%	8367	33.4%	10,245	38.2%
Obese	23,518	45.4%	11,865	47.4%	11,653	43.5%
Missing	1566	3.0%	1187	4.7%	379	1.4%
Employment						
Employed	13,254	25.6%	7150	28.6%	6104	22.8%
Retired	26,645	51.4%	10,900	43.6%	15,745	58.8%
Other	4415	8.5%	2658	10.6%	1757	6.6%
Missing	7489	14.5%	4307	17.2%	3182	11.9%
Ethnic group						
White	38,538	74.4%	17,144	68.5%	21,394	79.9%
Asian	6113	11.8%	3728	14.9%	2385	8.9%
Black	2952	5.7%	1597	6.4%	1355	5.1%
Mixed & other	1895	3.7%	1086	4.3%	809	3.0%
Missing	2305	4.4%	1460	5.8%	845	3.2%
Disability						
No disability	39,604	76.5%	18,561	74.2%	21,043	78.6%
Disability	8916	17.2%	4772	19.1%	4144	15.5%
Missing	3283	6.3%	1682	6.7%	1601	6.0%
Smoker						
Smoker	2991	5.8%	1885	7.5%	1106	4.1%
Ex-smoker	883	1.7%	400	1.6%	483	1.8%
Non-smoker	37,740	72.9%	16,809	67.2%	20,931	78.1%
Missing	10,189	19.7%	5921	23.7%	4268	15.9%

Characteristics recorded at referral or initial assessment, representing participants’ characteristics at the start of the programme. Programme completion is defined according to the NHS England definition of completion as attending at least 60% of sessions. *BMI* Body Mass Index

Individuals that completed the programme were on average older than those that only partially attended the programme. Whilst individuals aged over 65 represented 51.7% of those who only partially attended the programme, 64.3% of those completing the programme were in these age groups. Females represented 54.5% of total participants, and this was similar across the different participation groups.

Individuals that completed the programme were generally from less deprived neighbourhoods compared to those that did not. Of the individuals that partially attended the programme 19.0% lived in the most deprived quintile of areas in the country, compared to only 13.3% of individuals that completed the programme. Conversely, 21.7% of individuals that partially attended the programme were from the least deprived quintile, compared to 25.2% of individuals that completed the programme.

Amongst our main analysis sample of individuals attending at least one DPP session at the three providers with programmes of 13 sessions in length, 6.8% had developed type 2 diabetes by 31st March 2020 (Table 2). This figure was 8.6% among those who only partially attended the programme, and 5.1% amongst those who completed at least 60% of sessions. The raw rates of type 2 diabetes exhibited a downward gradient as attendance increased beyond six sessions, with 4% of those attending 12 or 13 sessions developing type 2 diabetes during the follow-up period.

**Table 2:** Descriptive statistics – Programme attendance and progression to type 2 diabetes

		N	%	Developed type 2 diabetes up to 31st March 2020	
				N	%
**Participants at the three providers with programmes of 13 sessions in length (*n* = 51,803)**					
	All participants	51,803	100%	3530	6.8%
Programme completion	Partial attendance (< 60% of sessions)	25,015	48.3%	2155	8.6%
	Completed programme (≥ 60% of sessions)	26,788	51.7%	1375	5.1%
Number of programme sessions attended	1 session	4391	8.5%	415	9.5%
	2 sessions	2976	5.7%	285	9.6%
	3 sessions	3412	6.6%	285	8.4%
	4 sessions	4238	8.2%	385	9.1%
	5 sessions	3409	6.6%	280	8.2%
	6 sessions	3347	6.5%	280	8.4%
	7 sessions	3242	6.3%	230	7.1%
	8 sessions	3216	6.2%	215	6.7%
	9 sessions	3492	6.7%	230	6.6%
	10 sessions	4231	8.2%	240	5.7%
	11 sessions	5490	10.6%	275	5.0%
	12 sessions	5847	11.3%	235	4.0%
	13 sessions	4512	8.7%	185	4.1%
**Participants at all four providers (*n* = 66,010)**					
	All participants	66,010	100%	4320	6.5%
Programme completion	Partial attendance (< 60% of sessions)	32,090	48.6%	2645	8.2%
	Completed programme (≥ 60% of sessions)	33,920	51.4%	1675	4.9%

Numbers of individuals diagnosed with type 2 diabetes are rounded to nearest five as per data sharing rules. Corresponding percentages are based on rounded numerators. Programme completion is defined according to the NHS England definition of completion as attending at least 60% of sessions

### Regression results

**Fig. 1:**
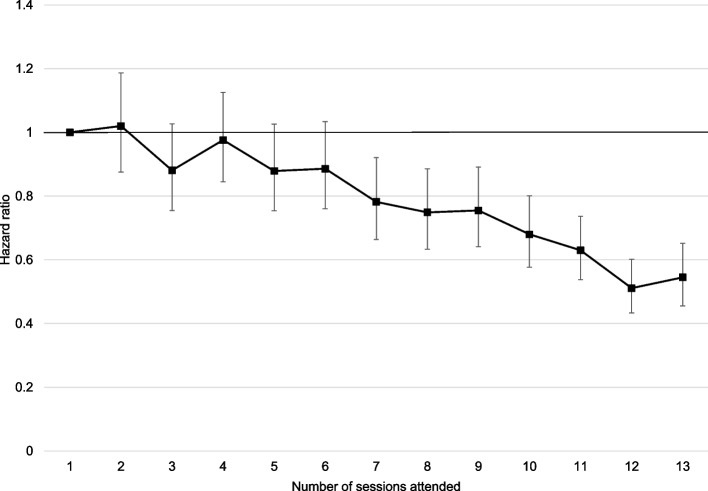
Hazard ratios from Weibull survival regressions of the risk of progression to type 2 diabetes and the number of sessions attended. Figure notes. Coefficient plot showing estimated hazard ratios from a Weibull survival regression of number of sessions attended and progression to type 2 diabetes by 31st March 2020 as presented in Table 3, column 2. Regressions include controls for participant characteristics

Table 3 and Fig. 1 present hazard ratios from the Weibull survival models of the association between number of sessions attended and type 2 diabetes diagnosis. There was no statistically significant difference in type 2 diabetes risk amongst individuals attending between two and six sessions relative to only attending one session (Table 3, column 2). Attending seven sessions and beyond was associated with a statistically significant lower type 2 diabetes risk compared to attending only one session, and the magnitude of this association increases with the number of sessions attended up to 12 sessions. Attending the full 13 sessions was associated with a 45.5% lower risk of developing type 2 diabetes relative to only attending one programme session (HR: 0.545 95% CI: 0.455 to 0.652). Table S1 of the additional materials presents the regression coefficients for all covariates included in the model.

**Table 3:** Weibull survival model of number of DPP sessions attended and risk of progression to type 2 diabetes

	Hazard ratio associated with progression to type 2 diabetes between 12 months from referral and 31st March 2020	
	Unadjusted	Adjusted
Attended 2 sessions	1.005	1.020
	[0.864,1.169]	[0.876,1.187]
Attended 3 sessions	0.880	0.881
	[0.756,1.023]	[0.755,1.027]
Attended 4 sessions	0.978	0.976
	[0.851,1.124]	[0.845,1.126]
Attended 5 sessions	0.849^*^	0.879
	[0.730,0.988]	[0.754,1.026]
Attended 6 sessions	0.842^*^	0.886
	[0.723,0.980]	[0.760,1.034]
Attended 7 sessions	0.723^***^	0.782^**^
	[0.615,0.849]	[0.664,0.921]
Attended 8 sessions	0.684^***^	0.749^***^
	[0.581,0.807]	[0.633,0.886]
Attended 9 sessions	0.663^***^	0.755^***^
	[0.565,0.780]	[0.641,0.891]
Attended 10 sessions	0.571^***^	0.680^***^
	[0.486,0.669]	[0.577,0.801]
Attended 11 sessions	0.504^***^	0.630^***^
	[0.433,0.587]	[0.538,0.737]
Attended 12 sessions	0.405^***^	0.511^***^
	[0.345,0.475]	[0.433,0.602]
Attended 13 sessions	0.419^***^	0.545^***^
	[0.352,0.498]	[0.455,0.652]
Observations	51,803	51,803

Coefficients are hazard ratios. 95% confidence intervals in brackets. The regression model in column 2 includes participant characteristics, coefficients are presented in additional materials Table S1. The regression model in column 2 also includes fixed effects for programme provider, NHS England Region and referral month. ^*^ *p* < 0.05, ^**^ *p* < 0.01, ^***^ *p* < 0.001

Table S2 of the additional materials presents the hazard ratios from the Weibull survival model of the association between the binary measure of DPP completion and type 2 diabetes diagnosis for both the main analysis sample of attenders at the three 13 session providers (column 1), and the wider sample of attenders at all four providers (column 2). The hazard ratio associated with programme completion (≥ 60% of sessions) relative to only partial programme attendance is 0.693 (95% CI: 0.645, 0.745) for participants at the three 13 session providers (column 1). This was of similar magnitude when we analysed participants at all four providers (HR: 0.686 95% CI: 0.643, 0.732) (column 2). Individuals that completed the DPP therefore had a 30.7% lower risk of type 2 diabetes compared with individuals that only attended part of the programme.

### Supplementary analysis

Table S3 of the additional materials presents the results when the at-risk period was specified as from the date of the first session onwards, as opposed to 12 months post-referral. There was no statistically significant difference in type 2 diabetes risk amongst individuals attending between two and five sessions relative to only attending one session. Attending six sessions and beyond was associated with a statistically significant lower type 2 diabetes risk compared to attending only one session. Attending the full 13 sessions was associated with a 50.5% lower risk of developing type 2 diabetes relative to only attending one programme session (HR: 0.495 95% CI: 0.419 to 0.585).

The results from the accelerated failure time regressions displayed similar patterns to those from the primary analysis (Table S4 of the additional materials). There was again no statistically significant difference in type 2 diabetes risk amongst individuals attending between two and six sessions relative to attending one session. Attending seven sessions and beyond was associated with a statistically significant lower type 2 diabetes risk compared to attending only one session, and the magnitude of this association was again found to increase with the number of sessions attended up to 12 sessions. We found that relative to attending only one programme session, attending the full 13 sessions was associated with a 78.5% slower time to type 2 diabetes diagnosis (time ratio (TR): 1.785 95% CI: 1.502 to 2.123).

## Discussion

### Summary of findings

The NHS DPP is an evidence based behavioural intervention designed to prevent individuals with non-diabetic hyperglycaemia developing type 2 diabetes. This is the first study to examine the association between the level of attendance at the DPP and the risk of progression to type 2 diabetes.

Attendance at a minimum of seven sessions, or 54% of the programme content, was required before a statistically significant reduction in type 2 diabetes risk was observed, relative to attending only one session. NHS England define programme completion as attending at least 60% of sessions, which is equivalent to 8 sessions for the providers that deliver 13 session programmes. Therefore, our findings suggest that this completion criterion is set at a level of attendance just over that required to achieve a demonstrated benefit. However, the magnitude of reduction in type 2 diabetes risk was found to increase up to attending 12 sessions, suggesting that there is potential for individuals to benefit further from attending more sessions than required under the current completion criteria. Individuals that attended the full 13 sessions had a 45.5% lower risk of type 2 diabetes, relative to attending only one session.

### Comparison to previous studies

Early outcomes analysis from the NHS DPP found a dose–response relationship between increased session attendance and intermediate outcomes of weight loss and HbA1c reduction [8]. They examined the association between the number of sessions attended, treated as a continuous variable, and outcomes amongst individuals that attended the initial assessment and at least one programme session. Each additional session attended was found to be associated with a -0.32kg greater weight loss and an additional 0.18 mmol/mol (0.02%) decrease in HbA1c. This analysis relied on outcomes measured by the programme providers during the programme, which were missing in a large proportion of attenders. Our analysis used outcomes captured in routinely collected primary care datasets and were therefore available for all participants. We examined the association between attendance and onset of type 2 diabetes, the reduction of which was the primary aim of the programme. Our findings confirmed that increased attendance at the NHS DPP was associated with a significant reduction in the targeted outcome of type 2 diabetes, detecting a dose–response relationship up to 12 sessions. This provides evidence that the previously detected dose–response relationship between session attendance and intermediate weight and HbA1c outcomes successfully translated into reductions in the targeted outcome of type 2 diabetes risk, confirming that the behavioural interventions remained effective when implemented at scale in routine care. However, we were not able to examine the mechanisms through which the intervention reduced diabetes risk in this paper, which remains an important area for future research.

A previous intention-to-treat analysis of the NHS DPP using data from a sample of general practices examined the effect of exposure to the DPP, defined as an individual having a recorded offer of the DPP in their primary care record [10]. The study found that those who were recorded as having been offered the NHS DPP were 20% less likely to develop type 2 diabetes compared to those who were not recorded as having been offered the programme. However, the primary care records were not able to confirm whether individuals who were recorded as having been offered the programme actually attended or completed the programme. Therefore, those estimates are diluted by individuals that were offered but did not take up the programme. Our study complements this analysis by examining the effect of attendance and completion amongst all individuals participating in the programme.

A study of the Norfolk Diabetes Prevention lifestyle intervention trial, the largest trial of a DPP outside the US DPP, found a 46% reduction in the risk of developing type 2 diabetes in the intervention compared to the control arm over an average of 24.7 months follow up [22]. They found significant reductions in intermediate outcomes such as mean HbA1c, weight and fasting plasma glucose between individuals that attended at least 60% of sessions compared to those who attended less than 30% of sessions. However, there was no significant difference in diabetes risk by level of attendance.

### Strengths and limitations

This is the first analysis of the effect of attendance and completion of a nationwide diabetes prevention programme on the risk of progression to type 2 diabetes. Assessing type 2 diabetes risk rather than intermediate outcome measures such as weight loss is relatively rare in the published literature. A meta-analysis evaluating real-world translational studies from the US DPP found that none of the 28 studies assessed changes in diabetes incidence [23].

We used rich nationally representative datasets on participants in the NHS DPP during the first two years of the programme, and all diagnoses of type 2 diabetes up to March 2020. This allowed us to link programme attendance and type 2 diabetes outcomes at the individual level. Our findings were robust to a range of sensitivity analyses.

The type 2 diabetes diagnosis outcome data relies on the identification and recording of diagnoses made within routine primary care. This is unlike the analysis of clinical trials of such programmes where outcomes will be collected at the trial follow-up intervals. It is possible therefore that not all instances of type 2 diabetes are captured, or that they may be observed later than in a trial setting. In addition, data are not available on deaths or migration outside England.

It is possible for individuals to be referred to the DPP more than once, with each referral representing a separate entry into the Minimum Data Set. However, since this referrals data are anonymised, we were unable to identify individuals and therefore multiple referrals for the same individual. Therefore, it is possible we observe the same person participating in the programme more than once.

The referral of individuals to the DPP and their level of programme attendance is not random. There are many ways individuals can be recruited into the programme, each associated with potential sources of selection bias. General practices decide if and when to start engaging with and referring to the DPP, and also which individuals to offer a DPP referral to. Following this, individuals then self-select into taking up this offer and their subsequent level of programme attendance. This study compared the effectiveness of the level of session attendance amongst individuals that decided to participate in the programme. We did not compare to individuals that did not take up the programme as their motivation and health-related behaviours are likely to differ significantly from those that decided to take up the programme. Comparing only among programme participants may therefore have helped reduce the extent of residual confounding within our analysis. However, comparing outcomes amongst attenders to individuals that did not take up the programme is an important area of future research.

The measured participant characteristics differed between individuals with different levels of attendance. Restricting our analysis to participants that participated in the programme allowed us to control for a richer set of individual characteristics for which data was only collected at the initial assessment (participant ethnicity, BMI at initial assessment, employment status, smoking status, and disability) and reduced the extent of residual confounding. Matching methods were inappropriate for our analysis due to the multiple levels of exposure and the large number of covariates and dimensions within them. As expected, controlling for the rich set of measured individual characteristics using regression adjustment resulted in lower effect estimates. However, it is possible that there is still residual confounding because we were only able to control for BMI and smoking status. There may be other behaviours prior to the start of the programme that are correlated with the subsequent number of sessions attended, such as sleep, physical activity, and sedentary behaviour.

## Conclusions

Reducing the risk of progression to type 2 diabetes through prevention programmes requires a minimum level of attendance. In the NHS DPP, attendance at a minimum of 7 of the 13 programme sessions, equivalent to 54% of programme content, was necessary before significant benefits were realised in terms of reduced diabetes risk. Retention of participants beyond this minimum level was associated with further benefits in diabetes risk reduction. However, very few individuals attended the full offer of programme sessions where the largest reductions in diabetes risk were seen. Whilst programme uptake is exceeding previously modelled estimates (24), our findings suggest further efforts should be made to encourage attendance and retention on the programme to further prevent cases of type 2 diabetes.

DPP providers are paid when participants reach set retention milestones. During the period we examined these payment milestones differed across the four providers, meaning that a minimum level of attendance at between 67 and 85% of programme sessions was required in order for all payment milestones to be met for a participant. This has since changed, with the overall stringency of the milestones relaxed and standardised across all providers. DPP providers can now receive the maximum payment if an individual attends eight of the 13 programme sessions, corresponding to 62% of sessions. Given the dose–response relationship we observe between session attendance and type 2 diabetes risk, commissioners may wish to consider altering the provider payment schedule to incentivise higher retention levels beyond 60% of programme sessions.

### Supplementary Information

Additional file 1: **Table S1.** Weibull survival model of number of DPP sessions attended and risk of progression to type 2 diabetes, presenting coefficients for all variables in models. **Table S2.** Survival models of programme completion and risk of progression to type 2 diabetes. **Table S3.** Supplementary analysis 1: association between number of sessions attended and type 2 diabetes when time at risk is defined as starting from the first session attended. **Table S4.** Supplementary analysis 2: accelerated failure time regression models for number of sessions attended and risk of progression to type 2 diabetes. 

## Data Availability

The data that support the findings of this study are available from NHS England and NHS Digital, but restrictions apply to the availability of these data, which were used under license for the current study and therefore are not publicly available. The authors are not permitted to share these data.

## Abbreviations

BMI: Body mass
CI: Confidence interval
DPP: Diabetes prevention programme
HR: Hazard ratio
NHS: National health service
TR: Time ratio
