# Supplementary material for: Level of attendance at the English National Health Service Diabetes Prevention Programme and risk of progression to type 2 diabetes
Source: Int J Behav Nutr Phys Act. 2024 Jan 12;21:6. doi: 10.1186/s12966-023-01554-7 (PMC10785516; doi:10.1186/s12966-023-01554-7)
Supplement: Supplementary file 1 — Additional file 1: Table S1. Weibull survival model of number of DPP sessions attended and risk of progression to type 2 diabetes, presenting coefficients for all variables in models. Table S2. Survival models of programme completion and risk of progression to type 2 diabetes. Table S3. Supplementary analysis 1: association between number of sessions attended and type 2 diabetes when time at risk is defined as starting from the first session attended. Table S4. Supplementary analysis 2: accelerated failure time regression models for number of sessions attended and risk of progression to type 2 diabetes. [file 12966_2023_1554_MOESM1_ESM.docx]

Table S1. Weibull survival model of number of DPP sessions attended and risk of progression to type 2 diabetes, presenting coefficients for all variables in models.

|  | Hazard ratio associated with progression to type 2 diabetes between 12 months from referral and 31^st^ March 2020 | |
| --- | --- | --- |
|  | Unadjusted | Adjusted |
| Attended 2 sessions | 1.005 | 1.020 |
|  | [0.864,1.169] | [0.876,1.187] |
|  |  |  |
| Attended 3 sessions | 0.880 | 0.881 |
|  | [0.756,1.023] | [0.755,1.027] |
|  |  |  |
| Attended 4 sessions | 0.978 | 0.976 |
|  | [0.851,1.124] | [0.845,1.126] |
|  |  |  |
| Attended 5 sessions | 0.849^*^ | 0.879 |
|  | [0.730,0.988] | [0.754,1.026] |
|  |  |  |
| Attended 6 sessions | 0.842^*^ | 0.886 |
|  | [0.723,0.980] | [0.760,1.034] |
|  |  |  |
| Attended 7 sessions | 0.723^***^ | 0.782^**^ |
|  | [0.615,0.849] | [0.664,0.921] |
|  |  |  |
| Attended 8 sessions | 0.684^***^ | 0.749^***^ |
|  | [0.581,0.807] | [0.633,0.886] |
|  |  |  |
| Attended 9 sessions | 0.663^***^ | 0.755^***^ |
|  | [0.565,0.780] | [0.641,0.891] |
|  |  |  |
| Attended 10 sessions | 0.571^***^ | 0.680^***^ |
|  | [0.486,0.669] | [0.577,0.801] |
|  |  |  |
| Attended 11 sessions | 0.504^***^ | 0.630^***^ |
|  | [0.433,0.587] | [0.538,0.737] |
|  |  |  |
| Attended 12 sessions | 0.405^***^ | 0.511^***^ |
|  | [0.345,0.475] | [0.433,0.602] |
|  |  |  |
| Attended 13 sessions | 0.419^***^ | 0.545^***^ |
|  | [0.352,0.498] | [0.455,0.652] |
| Age group: |  |  |
| Aged 18 to 34 |  | - |
|  |  | - |
|  |  |  |
| Aged 35 to 44 |  | 1.146 |
|  |  | [0.851,1.543] |
|  |  |  |
| Aged 45 to 54 |  | 1.218 |
|  |  | [0.918,1.616] |
|  |  |  |
| Aged 55 to 64 |  | 1.004 |
|  |  | [0.758,1.331] |
|  |  |  |
| Aged 65 to 74 |  | 0.841 |
|  |  | [0.630,1.124] |
|  |  |  |
| Aged 75 to 84 |  | 0.749 |
|  |  | [0.555,1.011] |
|  |  |  |
| Aged 85+ |  | 0.592^*^ |
|  |  | [0.397,0.883] |
|  |  |  |
| Male |  | 1.133^***^ |
|  |  | [1.059,1.213] |
| Deprivation quintile: |  |  |
| Most deprived |  | - |
|  |  | - |
|  |  |  |
| 2 |  | 0.876^*^ |
|  |  | [0.789,0.972] |
|  |  |  |
| 3 |  | 0.834^***^ |
|  |  | [0.750,0.927] |
|  |  |  |
| 4 |  | 0.807^***^ |
|  |  | [0.723,0.901] |
|  |  |  |
| Least deprived |  | 0.737^***^ |
|  |  | [0.657,0.828] |
|  |  |  |
| Missing |  | 1.801 |
|  |  | [0.805,4.033] |
| BMI: |  |  |
| Under/healthy weight |  | - |
|  |  | - |
|  |  |  |
| Overweight |  | 1.355^***^ |
|  |  | [1.189,1.544] |
|  |  |  |
| Obese |  | 2.340^***^ |
|  |  | [2.072,2.643] |
|  |  |  |
| Missing |  | 1.590^***^ |
|  |  | [1.273,1.987] |
| Employment category: |  |  |
| Employed |  | - |
|  |  | - |
|  |  |  |
| Retired |  | 1.104 |
|  |  | [0.993,1.228] |
|  |  |  |
| Other |  | 1.247^***^ |
|  |  | [1.109,1.402] |
|  |  |  |
| Missing |  | 1.204^**^ |
|  |  | [1.067,1.359] |
| Ethnicity: |  |  |
| White |  | - |
|  |  | - |
|  |  |  |
| Asian |  | 1.313^***^ |
|  |  | [1.183,1.457] |
|  |  |  |
| Black |  | 0.806^**^ |
|  |  | [0.690,0.941] |
|  |  |  |
| Mixed & Other ethnic groups |  | 1.210^*^ |
|  |  | [1.030,1.423] |
|  |  |  |
| Missing |  | 1.072 |
|  |  | [0.900,1.277] |
| Disability: |  |  |
| No disability |  | - |
|  |  | - |
|  |  |  |
| Disability |  | 1.234^***^ |
|  |  | [1.134,1.343] |
|  |  |  |
| Missing |  | 0.972 |
|  |  | [0.834,1.132] |
| Smoking status: |  |  |
| Smoker |  | - |
|  |  | - |
|  |  |  |
| Ex-smoker |  | 1.012 |
|  |  | [0.714,1.435] |
|  |  |  |
| Non-smoker |  | 0.983 |
|  |  | [0.860,1.122] |
|  |  |  |
| Missing |  | 0.899 |
|  |  | [0.767,1.053] |
| Observations | 51803 | 51803 |

Coefficients are hazard ratios. 95% confidence intervals in brackets. The regression model in column 2 also includes fixed effects for programme provider, NHS England Region, and referral month. BMI: Body Mass Index. ^*^ *p* < 0.05, ^**^ *p* < 0.01, ^***^ *p* < 0.001

Table S2. Survival models of programme completion and risk of progression to type 2 diabetes.

|  | Hazard ratio associated with progression to type 2 diabetes between 12 months from referral and 31^st^ March 2020 | |
| --- | --- | --- |
|  | Participants at 13 session providers | Participants at all providers |
| Completed programme (≥60% of sessions) | 0.693^***^ | 0.686^***^ |
|  | [0.645,0.745] | [0.643,0.732] |
| Age group: |  |  |
| Aged 18 to 34 | - | - |
|  | - | - |
|  |  |  |
| Aged 35 to 44 | 1.144 | 1.055 |
|  | [0.850,1.540] | [0.817,1.361] |
|  |  |  |
| Aged 45 to 54 | 1.217 | 1.091 |
|  | [0.917,1.615] | [0.857,1.390] |
|  |  |  |
| Aged 55 to 64 | 0.993 | 0.903 |
|  | [0.749,1.316] | [0.710,1.148] |
|  |  |  |
| Aged 65 to 74 | 0.828 | 0.763^*^ |
|  | [0.620,1.106] | [0.597,0.976] |
|  |  |  |
| Aged 75 to 84 | 0.741^*^ | 0.655^**^ |
|  | [0.549,0.999] | [0.507,0.846] |
|  |  |  |
| Aged 85+ | 0.593^*^ | 0.579^**^ |
|  | [0.398,0.884] | [0.410,0.818] |
|  |  |  |
| Male | 1.132^***^ | 1.144^***^ |
|  | [1.057,1.212] | [1.076,1.216] |
| Deprivation quintile: |  |  |
| Most deprived | - | - |
|  | - | - |
|  |  |  |
| 2 | 0.869^**^ | 0.893^*^ |
|  | [0.783,0.965] | [0.813,0.981] |
|  |  |  |
| 3 | 0.825^***^ | 0.847^***^ |
|  | [0.742,0.918] | [0.769,0.933] |
|  |  |  |
| 4 | 0.798^***^ | 0.837^***^ |
|  | [0.715,0.890] | [0.758,0.925] |
|  |  |  |
| Least deprived | 0.728^***^ | 0.765^***^ |
|  | [0.648,0.817] | [0.689,0.849] |
|  |  |  |
| Missing | 1.752 | 0.831 |
|  | [0.782,3.924] | [0.413,1.670] |
| BMI: |  |  |
| Under/normal weight | - | - |
|  | - | - |
|  |  |  |
| Overweight | 1.357^***^ | 1.352^***^ |
|  | [1.191,1.546] | [1.199,1.523] |
|  |  |  |
| Obese | 2.344^***^ | 2.340^***^ |
|  | [2.075,2.647] | [2.093,2.616] |
|  |  |  |
| Missing | 1.640^***^ | 1.684^***^ |
|  | [1.316,2.045] | [1.441,1.968] |
| Employment category: |  |  |
| Employed | - | - |
|  | - | - |
|  |  |  |
| Retired | 1.097 | 1.097 |
|  | [0.986,1.220] | [0.996,1.208] |
|  |  |  |
| Other | 1.250^***^ | 1.257^***^ |
|  | [1.112,1.405] | [1.124,1.404] |
|  |  |  |
| Missing | 1.199^**^ | 1.156^**^ |
|  | [1.063,1.353] | [1.040,1.284] |
| Ethnicity: |  |  |
| White | - | - |
|  | - | - |
|  |  |  |
| Asian | 1.325^***^ | 1.347^***^ |
|  | [1.194,1.470] | [1.224,1.482] |
|  |  |  |
| Black | 0.805^**^ | 0.807^**^ |
|  | [0.689,0.941] | [0.702,0.927] |
|  |  |  |
| Mixed & Other ethnic groups | 1.219^*^ | 1.198^*^ |
|  | [1.037,1.432] | [1.030,1.394] |
|  |  |  |
| Missing | 1.080 | 0.969 |
|  | [0.907,1.286] | [0.847,1.110] |
| Disability: |  |  |
| No disability | - | - |
|  | - | - |
|  |  |  |
| Disability | 1.240^***^ | 1.254^***^ |
|  | [1.140,1.350] | [1.157,1.359] |
|  |  |  |
| Missing | 0.972 | 1.002 |
|  | [0.835,1.132] | [0.889,1.129] |
| Smoking status: |  |  |
| Smoker | - | - |
|  | - | - |
|  |  |  |
| Ex-smoker | 1.003 | 1.048 |
|  | [0.708,1.423] | [0.764,1.438] |
|  |  |  |
| Non-smoker | 0.968 | 0.979 |
|  | [0.848,1.105] | [0.861,1.112] |
|  |  |  |
| Missing | 0.885 | 0.906 |
|  | [0.755,1.036] | [0.782,1.049] |
| Observations | 51803 | 66010 |

Coefficients are hazard ratios. 95% confidence intervals in brackets. Models also include fixed effects for provider, NHS England Region, and referral month. BMI: Body Mass Index. ^*^ *p* < 0.05, ^**^ *p* < 0.01, ^***^ *p* < 0.001

Table S3. Supplementary analysis 1: association between number of sessions attended and type 2 diabetes when time at risk is defined as starting from the first session attended.

|  | Hazard ratio of progression to type 2 diabetes between first session and 31^st^ March 2020 |
| --- | --- |
| Attended 2 sessions | 1.027 |
|  | [0.895,1.178] |
|  |  |
| Attended 3 sessions | 0.927 |
|  | [0.808,1.064] |
|  |  |
| Attended 4 sessions | 0.987 |
|  | [0.868,1.123] |
|  |  |
| Attended 5 sessions | 0.873 |
|  | [0.758,1.004] |
|  |  |
| Attended 6 sessions | 0.834^*^ |
|  | [0.723,0.961] |
|  |  |
| Attended 7 sessions | 0.785^**^ |
|  | [0.677,0.912] |
|  |  |
| Attended 8 sessions | 0.738^***^ |
|  | [0.634,0.860] |
|  |  |
| Attended 9 sessions | 0.731^***^ |
|  | [0.628,0.849] |
|  |  |
| Attended 10 sessions | 0.666^***^ |
|  | [0.574,0.773] |
|  |  |
| Attended 11 sessions | 0.607^***^ |
|  | [0.525,0.701] |
|  |  |
| Attended 12 sessions | 0.486^***^ |
|  | [0.418,0.566] |
|  |  |
| Attended 13 sessions | 0.495^***^ |
|  | [0.419,0.585] |
| Age group: |  |
| Aged 18 to 34 | - |
|  | - |
|  |  |
| Aged 35 to 44 | 1.096 |
|  | [0.841,1.427] |
|  |  |
| Aged 45 to 54 | 1.150 |
|  | [0.895,1.477] |
|  |  |
| Aged 55 to 64 | 0.955 |
|  | [0.744,1.226] |
|  |  |
| Aged 65 to 74 | 0.817 |
|  | [0.632,1.056] |
|  |  |
| Aged 75 to 84 | 0.723^*^ |
|  | [0.555,0.943] |
|  |  |
| Aged 85+ | 0.659^*^ |
|  | [0.467,0.930] |
|  |  |
| Male | 1.083^*^ |
|  | [1.018,1.152] |
| Deprivation quintile: |  |
| Most deprived | - |
|  | - |
|  |  |
| 2 | 0.888^*^ |
|  | [0.808,0.977] |
|  |  |
| 3 | 0.848^***^ |
|  | [0.770,0.934] |
|  |  |
| 4 | 0.855^**^ |
|  | [0.774,0.945] |
|  |  |
| Least deprived | 0.768^***^ |
|  | [0.692,0.853] |
|  |  |
| Missing | 1.346 |
|  | [0.607,2.982] |
| BMI: |  |
| Under/normal weight | - |
|  | - |
|  |  |
| Overweight | 1.325^***^ |
|  | [1.177,1.492] |
|  |  |
| Obese | 2.306^***^ |
|  | [2.065,2.576] |
|  |  |
| Missing | 1.674^***^ |
|  | [1.373,2.042] |
| Employment category: |  |
| Employed | - |
|  | - |
|  |  |
| Retired | 1.070 |
|  | [0.971,1.180] |
|  |  |
| Other | 1.278^***^ |
|  | [1.149,1.421] |
|  |  |
| Missing | 1.159^**^ |
|  | [1.039,1.293] |
| Ethnicity: |  |
| White | - |
|  | - |
|  |  |
| Asian | 1.260^***^ |
|  | [1.145,1.386] |
|  |  |
| Black | 0.786^**^ |
|  | [0.680,0.908] |
|  |  |
| Mixed & Other ethnic groups | 1.184^*^ |
|  | [1.021,1.372] |
|  |  |
| Missing | 1.066 |
|  | [0.909,1.249] |
| Disability: |  |
| No disability | - |
|  | - |
|  |  |
| Disability | 1.241^***^ |
|  | [1.149,1.340] |
|  |  |
| Missing | 0.989 |
|  | [0.862,1.135] |
| Smoking status: |  |
| Smoker | - |
|  | - |
|  |  |
| Ex-smoker | 1.097 |
|  | [0.817,1.472] |
|  |  |
| Non-smoker | 0.945 |
|  | [0.839,1.064] |
|  |  |
| Missing | 0.957 |
|  | [0.831,1.104] |
| Observations | 52390 |

Coefficients are hazard ratios. 95% confidence intervals in brackets. Models also include fixed effects for provider, NHS England Region, and referral month. BMI: Body Mass Index ^*^ *p* < 0.05, ^**^ *p* < 0.01, ^***^ *p* < 0.001

Table S4. Supplementary analysis 2: accelerated failure time regression models for number of sessions attended and risk of progression to type 2 diabetes

|  | Time ratio of progression to type 2 diabetes by 31^st^ March 2020 |
| --- | --- |
| Attended 2 sessions | 0.982 |
|  | [0.849,1.135] |
|  |  |
| Attended 3 sessions | 1.129 |
|  | [0.974,1.307] |
|  |  |
| Attended 4 sessions | 1.024 |
|  | [0.893,1.174] |
|  |  |
| Attended 5 sessions | 1.130 |
|  | [0.975,1.310] |
|  |  |
| Attended 6 sessions | 1.122 |
|  | [0.968,1.300] |
|  |  |
| Attended 7 sessions | 1.265^**^ |
|  | [1.081,1.480] |
|  |  |
| Attended 8 sessions | 1.318^***^ |
|  | [1.122,1.549] |
|  |  |
| Attended 9 sessions | 1.307^***^ |
|  | [1.116,1.530] |
|  |  |
| Attended 10 sessions | 1.446^***^ |
|  | [1.236,1.691] |
|  |  |
| Attended 11 sessions | 1.556^***^ |
|  | [1.337,1.810] |
|  |  |
| Attended 12 sessions | 1.899^***^ |
|  | [1.619,2.228] |
|  |  |
| Attended 13 sessions | 1.785^***^ |
|  | [1.502,2.123] |
| Age group: |  |
| Aged 18 to 34 | - |
|  | - |
|  |  |
| Aged 35 to 44 | 0.878 |
|  | [0.661,1.166] |
|  |  |
| Aged 45 to 54 | 0.828 |
|  | [0.632,1.085] |
|  |  |
| Aged 55 to 64 | 0.996 |
|  | [0.761,1.303] |
|  |  |
| Aged 65 to 74 | 1.179 |
|  | [0.895,1.555] |
|  |  |
| Aged 75 to 84 | 1.317 |
|  | [0.990,1.753] |
|  |  |
| Aged 85+ | 1.650^*^ |
|  | [1.126,2.417] |
|  |  |
| Male | 0.887^***^ |
|  | [0.831,0.947] |
| Deprivation quintile: |  |
| Most deprived | - |
|  | - |
|  |  |
| 2 | 1.135^*^ |
|  | [1.027,1.254] |
|  |  |
| 3 | 1.189^***^ |
|  | [1.075,1.316] |
|  |  |
| 4 | 1.227^***^ |
|  | [1.105,1.363] |
|  |  |
| Least deprived | 1.338^***^ |
|  | [1.198,1.495] |
|  |  |
| Missing | 0.570 |
|  | [0.264,1.231] |
| BMI: |  |
| Under/normal weight | - |
|  | - |
|  |  |
| Overweight | 0.748^***^ |
|  | [0.660,0.847] |
|  |  |
| Obese | 0.444^***^ |
|  | [0.394,0.500] |
|  |  |
| Missing | 0.642^***^ |
|  | [0.519,0.795] |
| Employment category: |  |
| Employed | - |
|  | - |
|  |  |
| Retired | 0.910 |
|  | [0.822,1.007] |
|  |  |
| Other | 0.810^***^ |
|  | [0.724,0.906] |
|  |  |
| Missing | 0.838^**^ |
|  | [0.746,0.940] |
| Ethnicity: |  |
| White | - |
|  | - |
|  |  |
| Asian | 0.771^***^ |
|  | [0.698,0.852] |
|  |  |
| Black | 1.229^**^ |
|  | [1.059,1.426] |
|  |  |
| Mixed & Other ethnic groups | 0.833^*^ |
|  | [0.714,0.973] |
|  |  |
| Missing | 0.936 |
|  | [0.792,1.105] |
| Disability: |  |
| No disability | - |
|  | - |
|  |  |
| Disability | 0.818^***^ |
|  | [0.754,0.887] |
|  |  |
| Missing | 1.028 |
|  | [0.889,1.189] |
| Smoking status: |  |
| Smoker | - |
|  | - |
|  |  |
| Ex-smoker | 0.989 |
|  | [0.708,1.380] |
|  |  |
| Non-smoker | 1.017 |
|  | [0.896,1.154] |
|  |  |
| Missing | 1.108 |
|  | [0.952,1.288] |
| Observations | 51803 |

Coefficients are time ratios. 95% confidence intervals in brackets. Models also include fixed effects for provider, NHS England Region, and referral month. BMI: Body Mass Index. ^*^ *p* < 0.05, ^**^ *p* < 0.01, ^***^ *p* < 0.001
